# Temporal plasticity in habitat selection criteria explains patterns of animal dispersal

**DOI:** 10.1093/beheco/ary193

**Published:** 2019-01-12

**Authors:** Casey C Day, Nicholas P McCann, Patrick A Zollner, Jonathan H Gilbert, David M MacFarland

**Affiliations:** 1 Department of Forestry and Natural Resources, Purdue University, West Lafayette, IN, USA; 2 Great Lakes Indian Fish and Wildlife Commission, Odanah, WI, USA; 3 Wisconsin Department of Natural Resources, Rhinelander, WI, USA

**Keywords:** habitat specificity, home range, individual-based model IBM, *Martes americana*, movement ecology, pattern-oriented modeling

## Abstract

Patterns of dispersal behavior are often driven by the composition and configuration of suitable habitat in a matrix of unsuitable habitat. Interactions between animal behavior and landscapes can therefore influence population dynamics, population and species distributions, population genetic structure, and the evolution of behavior. Spatially explicit individual-based models (IBMs) are ideal tools for exploring the effects of landscape structure on dispersal. We developed an empirically parameterized IBM in the modeling framework SEARCH to simulate dispersal of translocated American martens in Wisconsin. We tested the hypothesis that a time-limited disperser should be willing to settle in lower quality habitat over time. To evaluate model performance, we used a pattern-oriented modeling approach. Our best model matched all empirical dispersal patterns (e.g., dispersal distance) except time to settlement. This model incorporated a required search phase as well as the mechanism for declining habitat selectivity over time, which represents the first demonstration of this hypothesis for a vertebrate species. We suggest that temporal plasticity in habitat selectivity allows individuals to maximize fitness by making a tradeoff between habitat quality and risk of mortality. Our IBM is pragmatic in that it addresses a management need for a species of conservation concern. However, our model is also paradigmatic in that we explicitly tested a theory of dispersal behavior. Linking these 2 approaches to ecological modeling can further the utility of individual-based modeling and provide direction for future theoretical and empirical work on animal behavior.

## Teaser

Home range selection is a complex task for animal dispersers. Using a simulation modeling approach, we created virtual American martens whose dispersal patterns matched those of actual martens. The best-performing model included martens that were willing to settle in lower quality habitat over time and searched for at least 2 weeks prior to settlement. We demonstrate how such a modeling approach can simultaneously answer questions that are of a theoretical and an applied nature.

## INTRODUCTION

Interactions between landscape-level patterns and animal decision-making behaviors can regulate important ecological and evolutionary processes for both species and communities (Lima and Zollner 1996). For example, much research has focused on how patterns of animal movement are affected by the composition and configuration of landscapes (Schick et al. 2008). Animals often adjust the speed and/or straightness of their movement as a result of factors such as vegetation type (Roshier et al. 2008), disturbance (Anadón et al. 2012), and patchiness (Johnson et al. 2001). These fine-scale effects may ultimately drive major demographic or ecological processes including invasions of non-native species (Holway and Suarez 1999) or spatial and temporal variations in foraging behavior (Johnson et al. 2001; Launchbaugh and Howery 2005). Likewise, interactions between animal movement and landscape structure can shape the connectivity and distribution of populations through dispersal and settlement (Bowler and Benton 2005; Burgess et al. 2012).

Animal dispersal comprises a complex series of events that begin with the decision to emigrate from one’s current habitat patch and end with immigration to a new settlement patch. Between emigration and immigration, individuals undergo an exploratory phase wherein they travel among habitat patches in search of a suitable home range (Bowler and Benton 2005). Home range selection is critical to the long-term survival of the individual because the home range provides essential benefits (e.g., food and cover) that ideally outweigh daily maintenance costs (Powell 2000). Dispersal behavior is therefore critical to the long-term survival of the individual, and also to the demographic and genetic viability of the population. Benefits of successful dispersal include inbreeding avoidance, increases in individual fitness, range (and resource) expansion, and genetic and demographic rescue, among others (Bowler and Benton 2005). Given the importance of dispersal to animal populations, one would expect individuals to employ dispersal strategies that maximize their likelihood of success.

The ability to understand and evaluate the complexities of animal dispersal can have important implications for the conservation and management of animal populations. However, describing these strategies has proven difficult, likely because animal behavior is driven by underlying decision-making processes and latent behavioral states that are more difficult to describe than the behaviors themselves (Nathan et al. 2008; Lichti et al. 2017). Classic models of dispersal assumed that individuals would distribute themselves according to an Ideal Free Distribution, whereby distribution would be directly correlated with resource availability (Fretwell and Calver 1970; Fretwell 1972). Subsequent work identified additional factors that affect the quality and timing of home range selection including natal experience (Mabry and Stamps 2008), body size (Stamps 1988), and conspecific attraction (Muller et al. 1997). Such work has improved our understanding of the processes that regulate dispersal and home range selection.

Another interaction that has the potential to affect dispersal behavior exists between the timing of home range establishment and the quality of the selected home range. Ward (1987) built on the experimental (Knight-Jones 1951, 1953; Wilson 1953) and theoretical (Levins 1968; Levins and MacArthur 1969; Jaenike 1978) work of others to propose a model of dispersal whereby selectivity in habitat selection declines over time for time-limited dispersers (i.e., dispersers with a finite amount of time to establish a home range). Under this model, the probability of selecting the best location for a home range is dependent on the likelihood of encountering and accepting similar (or better) habitat in the future. A suboptimal location that is initially rejected by the disperser may be accepted at a later time once some threshold of habitat selectivity is reached. This is in contrast to the null hypothesis that home range criteria remain static throughout the dispersal period. Ward’s (1987) hypothesis has received support from studies on the foraging, oviposition, and dispersal of larval marine invertebrates and insects [e.g., Qian (1999) and Toonen and Pawlik (2001)]. However, the hypothesis has not been tested with more behaviorally complex vertebrate species and has not been evaluated within spatially explicit frameworks. If the hypothesis accurately describes dispersal behavior, one would expect that the minimum threshold for what constitutes suitability would be inversely correlated with the amount of time required for dispersal.

Given the complexities of dispersal behavior, modeling the mechanisms that drive dispersal patterns can be challenging (Nathan 2001). Heterogeneity in habitat composition can significantly affect movement patterns during dispersal (Schultz and Crone 2001; Baguette and Van Dyck 2007), mortality risk and energy reserves can interact with habitat distribution to regulate dispersal success (Zollner and Lima 1999), and perceptual range can significantly affect an animal’s ability to detect suitable home range locations (Lima and Zollner 1996). Deliberate exploration behavior may also be undertaken prior to and during the dispersal process (Debeffe et al. 2013). Long-distance dispersal, a rare yet important component of dispersal behavior, is another factor that can be difficult to measure and describe (Trakhtenbrot et al. 2005). Individual-based models (IBMs) of animal dispersal—in which movements are simulated at the scale of the individual rather than the population—provide a mechanistic approach for reproducing complex dispersal behaviors. Because IBMs use simple, bottom-up rules to govern interactions of the disperser with the landscape and with conspecifics, mechanistic patterns of dispersal can emerge that are robust to alternative scenarios of landscape structure and resource distribution [e.g., Kramer-Schadt et al. (2011)]. Given this individual approach, IBMs are an ideal tool for capturing individual variation in animal movements that produce the complex patterns of dispersal observed in nature (Grimm et al. 2005).

We developed an empirically parameterized IBM of dispersal behavior of the American marten (*Martes americana*) in Wisconsin and tested the prediction by Ward (1987) that home range habitat requirements become less stringent throughout the dispersal period (i.e., declining habitat selectivity; Qian 1999). Martens are solitary carnivores that, like most weasel species, usually disperse from their natal grounds during the first year after birth (Johnson et al. 2009). In Wisconsin, their home ranges are associated with increasing canopy cover and complex forest structure (McCann et al. 2014). The marten represents an excellent case study for testing predictions of dispersal behavior, for several reasons. Martens are highly vagile and capable of long-distance dispersal events. They are also territorial and have specific habitat requirements, features that create unique interactions among dispersers and their environment (Powell 1979; Dumyahn et al. 2007). Furthermore, much is known about marten dispersal and habitat selection in our study area, as they have been the subject of conservation management programs since the 1970s (Wright 1999; Dumyahn et al. 2007; Williams et al. 2007; McCann et al. 2014). Despite continued efforts, it is possible that low survival and recruitment in some areas have resulted in a recovery that has been stagnant since the initial reintroduction of martens to northern Wisconsin (Woodford et al. 2005; McCann et al. 2010; Manlick et al. 2017). As dispersal and home range establishment are closely linked to juvenile survival, tools are needed that can be used to evaluate factors that may be influencing dispersal, habitat selection, and home range establishment.

Our primary objective was to evaluate alternative mechanisms for habitat selection of translocated martens in Northern Wisconsin. Specifically, we evaluated Ward’s hypothesis of habitat selection for its ability to explain patterns of marten dispersal behavior. We also tested whether a required exploratory phase improved the ability of our model to match empirical patterns of marten dispersal. To test these hypotheses, we developed an IBM of fine-scale marten movement with the goal of accurately simulating broad-scale patterns of dispersal behavior.

## METHODS

### Study system

**Figure 1:**
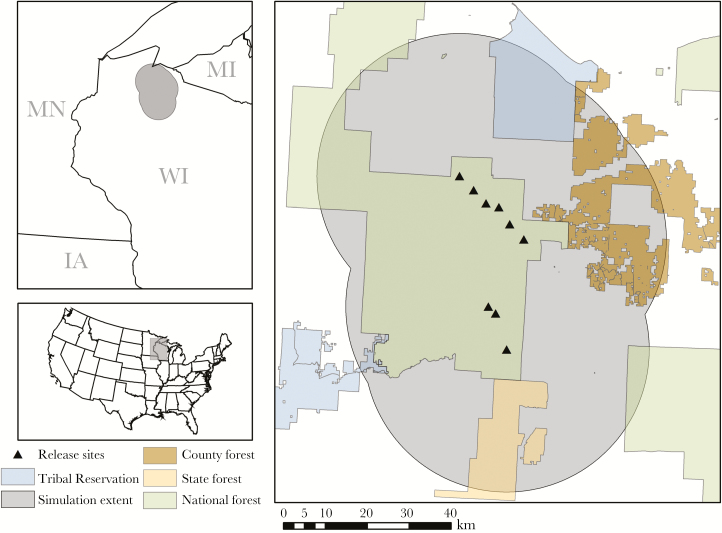
Study area in Northern Wisconsin, USA, where we simulated the dispersal of 15 translocated American martens in 2010.

We simulated the dispersal and home range establishment of 15 radio-collared martens translocated to the Chequamegon-Nicolet National Forest (CNNF) in North Central Wisconsin in 2010 (Figure 1). These releases were part of a larger augmentation that took place from 2008 to 2010 (Woodford 2010) and were in addition to 139 individuals translocated to the western CNNF in 1987–1990 as well as previous translocations to the eastern CNNF after extirpation of martens from Wisconsin by 1925 (Williams et al. 2007). To calibrate the model, data on mortality, dispersal, and home range establishment for the 15 translocated individuals were collected using radio-telemetry (Woodford et al. 2013) and compared with simulation results (Table 1).

**Table 1:** Patterns evaluated for calibration of a model of American marten dispersal following a translocation in Northern Wisconsin in 2010

Pattern	Hierarchical level	Weight	Observed pattern	Pass/Fail	Rank-sum	TI	D^2^
Dispersal distance mean	Individual	High	13.9 km	x	x	x	x
Dispersal distance SD	Population	Medium	13.2 km	x	x	x	
Days to HR establishment mean	Individual	Medium	37.3 days	x	x	x	x
Days to HR establishment SD	Population	Medium	10.3 days	x	x	x	
Average neighbor distance mean	Individual	High	26.4 km	x	x	x	x
Nearest neighbor distance mean	Individual	Medium	11.5 km	x	x	x	x
Mortality rate	Population	Low	0.17	x	x	x	
M:F Dispersal distance	Population	Low	M > F	x			
M:F Days to establishment	Population	Low	M > F	x			

An “x” in the last 4 columns indicates whether a given pattern was evaluated using that column’s method for pattern-matching analysis. All patterns were derived from empirical data from translocated martens in Northern Wisconsin. Hierarchical level indicates whether the pattern was averaged across individuals or was a characteristic of the population. Weight indicates the importance of each pattern as assigned by the authors. Pass/Fail represents a binary matching criterion and assigned points to a model based on whether a given pattern was successfully matched. Rank-sum ranked each model according to its ability to match each pattern and then summed the resulting ranks. TI (Total Indicator) ranked models based on their root mean square deviation from empirical patterns. *D*^2^ (Mahalanobis distance) ranked models based on a multivariate measure that accounts for covariance among patterns.

To define the extent of the simulation area, we extended marten release locations by 34 km, representing the upper 95% confidence limit of marten dispersal distance measured from martens released during an earlier translocation to the eastern CNNF (Davis 1983). Our resulting study area was 6956 km^2^ and contained portions of Ashland, Bayfield, Iron, Price, and Sawyer Counties. This area was comprised in large part by the CNNF and included significant portions of county forest, state land, tribal reservations, commercial/residential forested land, and urban/residential areas (Figure 1). Forests in the translocation areas were predominantly northern hardwoods dominated by sugar maple (*Acer saccharum*), red maple (*Acer rubrum*), and birch (*Betula* spp.). Stands of quaking aspen (*Populus tremuloides*) and mixed and conifer stands including balsam fir (*Abies balsamea*), white spruce (*Picea glauca*), northern white cedar (*Thuja occidentalis*), eastern hemlock (*Tsuga canadensis*), white pine (*Pinus strobus*), and red pine (*Pinus resinosa*) were common as well. Topography was generally flat, except for where the Gogebic range intersected the northwest portion of the study area, rising in some locations over 150 m to reach elevations greater than 570 m above sea level.

### Model description

To simulate the dispersal and home range establishment of translocated martens, we combined a habitat suitability model (Wright 1999, Dumyahn et al. 2007) with an IBM of animal dispersal [e.g., Kramer-Schadt et al. (2011) and Kanagaraj et al. (2013)] in the modeling framework Spatially Explicit Animal Response to Composition of Habitat (SEARCH; Pauli et al. 2013; Mutascio et al. 2017). In SEARCH, solitary dispersers move across a spatially explicit landscape, querying four independent, vector-based spatial layers that govern the following sets of parameters during each time step: food availability, predation risk, movement, and habitat suitability/sociality (see Supplementary Appendix S1 for a schematic of model processes). Each of these sets of parameters may vary both spatially and temporally. For example, food availability may vary spatially by vegetation type, but can also vary daily, seasonally, or annually. Dispersers navigate their environment while locating prey, minimizing predation risk, and searching for suitable habitat that is unoccupied by a conspecific of the same sex (Supplementary Appendix S1). When an area of contiguous suitable habitat ≥ 10% of the minimum home range size is encountered, the location is added to a list of potentially suitable locations stored in the individual’s memory. When the individual decides to attempt home range establishment, it sorts the existing list of stored locations based on a combination of factors including food availability, predation risk, and proximity to present location. The disperser then travels to the top-ranked site and attempts to establish a home range. If the amount of available (i.e., unoccupied) suitable habitat at that site is inadequate to support a home range, the individual either continues searching or reevaluates the list of sites and orients toward the new top priority. This process continues until the individual successfully establishes a home range, dies from predation or starvation, or dies at the end of the dispersal season (Supplementary Appendix S1).

In our application of SEARCH, we simulated a 60-day dispersal period divided into 5760 time steps of 15 min each. Martens actively dispersed for an average of 9.1 h per day (SD = 0.7), values that corresponded to activity periods in the fall in the same study area (Gilbert et al. 2009). Release locations and sex of the 15 dispersers were chosen based on actual marten releases in 2010. Except where noted, all other parameterizations were derived from data collected from a separate release of martens that were equipped with radio-collars in 2009. This allowed us to maintain independence between data used for parameterizing model agents and data used for calibration and pattern-matching. Parameterization of the 4 spatial layers (described below) followed methods used by McCann (2011).

### Spatial layers: habitat suitability

**Figure 2:**
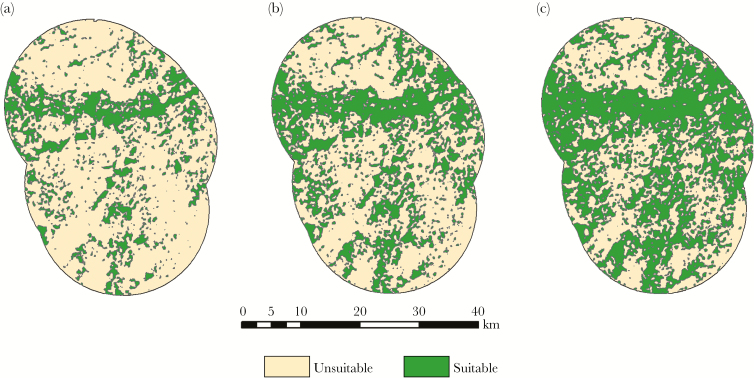
Examples of alternative rules used for habitat suitability in SEARCH modeling of American marten dispersal in Northern Wisconsin. Panels illustrate the dynamic progression of habitat selectivity over time from the 70% (a), to the 60% (b), and to the 50% (c) threshold rules. Percentages represent the proportion of an area around a pixel in the cover type data that must be represented by nonavoided cover types to be classified as suitable. Therefore, as the percentage decreases over time, martens perceive an increasing proportion of the landscape as suitable and habitat selectivity declines.

We defined habitat suitability according to the habitat model described by Wright (1999) and implemented this model in SEARCH as a binary layer of suitable/unsuitable habitat. Previous work in our study area demonstrated that at least 70% of marten home ranges were composed of preferred or neutral (i.e., nonavoided) habitat types (Bissonette et al. 1997; Wright 1999; Dumyahn et al. 2007). According to our habitat model (Wright 1999), suitability was dependent on both cover type (i.e., primary forest type) and size of the trees (i.e., Diameter at Breast Height) in each stand (Wright 1999). To determine cover type and tree size for public lands on our study area, we retrieved all available stand-level data within the simulation extent from the Wisconsin Forest Inventory and Reporting System (WisFIRS), the CNNF, and the Bad River Band of the Lake Superior Chippewa Tribe. These data included both the primary cover type (e.g., northern hardwood, lowland conifer, and upland conifer) and size of the trees in the stand. We then classified each of these stands as preferred, neutral, or avoided habitat types based on Wright’s (1999) classification. Preferred and neutral stands (i.e., nonavoided stands) received a suitability value of 1, whereas avoided habitat types received a suitability value of 0. For the proportion of the landscape for which we lacked stand data (48.8%), we used the remotely sensed Wisconsin Land Cover Data set (Wiscland) Level III. These data provided cover type but not tree size data. Because habitat suitability varied with tree size, we assigned habitat suitability values relative to the proportions of the corresponding stand-level data that were considered nonavoided. For example, 17% of aspen on public lands were nonavoided cover types (i.e., saw log class); therefore, pixels with the aspen cover type from the Wiscland data received a suitability value of 0.17. After assigning suitability values, we used the Focal Statistics tool in ArcGIS 10.3 (Esri, Redlands, CA) to identify pixels where 70% of the surrounding area in a 1 km^2^ buffer represented nonavoided cover types. To test Ward’s hypothesis of declining habitat selectivity over time, we adjusted the 70% value to represent varying degrees of habitat suitability as perceived by dispersing martens. The resulting rasters were then converted to vector as binary habitat suitability layers in order to be implemented in SEARCH (Figure 2).

Because martens are territorial, the spatial distribution of resident martens can influence dispersal behavior. We created a resident population of martens with home ranges using a combination of known and simulated resident locations. First, we populated an unoccupied landscape with 22 known home ranges based on 100% minimum convex polygons (JH Gilbert unpublished data; Dumyahn et al. 2007). To populate the rest of the landscape with resident home ranges, we simulated the release and home range establishment of an additional 78 martens based on a pretranslocation population estimate of 100 martens for the study area (Figure 2; Gilbert JH, unpublished data). Release locations for these simulated residents were distributed randomly throughout suitable habitat and individuals were allowed to establish a home range immediately upon release. We conducted these randomized establishment runs for each alternative model of habitat suitability.

### Spatial layers: movement

Inputs that informed the movement layer included distance moved per time step (mean step length, MSL), average turning angle per time step (expressed as mean vector length, MVL; Benhamou, 2004), perceptual range, and probability of crossing between vegetation types. We calculated MSL and MVL for each cover type by snow-trailing martens on the study area during winters 2008–2009 and 2009–2010 [see McCann et al. (2014) for a full treatment of these data]. These values differed by cover type and by sex (Moriarty et al. 2016). Females averaged shorter MSL and sharper turning angles than males, and we calculated the female:male ratios to be 0.9:1 for MSL and 0.97:1 for MVL. We set perceptual distance at 100 m for all cover types (Gardner and Gustafson 2004). We assumed that dispersing martens crossed freely between forested cover types, but rarely (with a 1% probability) crossed into unforested cover types (e.g., clearcuts, lakes, and urban areas; Chapin et al. 1998; Hargis et al. 1999; Moriarty et al. 2016).

### Spatial layers: food availability

Inputs that informed the food availability layer included probability of prey capture and mean (±SD) energy acquired given a successful predation event. Each of these parameters varied spatially according to the cover type occupied by the disperser in each time step. This layer affected dispersal because failure to locate prey over time could result in starvation and thus a failed dispersal attempt. Food availability was also considered when a marten was selecting among multiple potential home range locations. We calculated probability of prey capture and species of prey acquisition from the previously described snow-trailing data (McCann et al. 2014). Mean energy acquired (kJ) from a predation event was derived from estimates reported by Cumberland et al. (2001). Martens began each simulation with an initial energy reserve of 5491 kJ, calculated by multiplying the average mass of a marten by the average fraction of a marten composed of fat (Gilbert et al. 2009). Martens expended 10.5 kJ of energy per time step (1006 kJ/day; Gilbert et al. 2009). Individuals died from starvation after energy reserves reached 0 kJ.

### Spatial layers: predation risk

The predation risk layer also varied spatially by cover type. The only parameter associated with the predation risk layer was probability of mortality due to predation. We calculated this probability per time step based on mortality data collected from an earlier translocation during which martens were monitored for an average of 70 days on the CNNF (Davis 1983). We allocated risk to each cover type based on indices of relative abundance of the primary predators of martens (i.e., fishers [*Pekania pennanti*] and owls; McCann et al. 2010). We calculated these indices based on the presence of fisher tracks (McCann et al. 2014) and relevant owl calls (PAZ, unpublished data) in each cover type. We distributed unknown causes of mortality equally across all cover types.

### Study design

**Figure 3:**
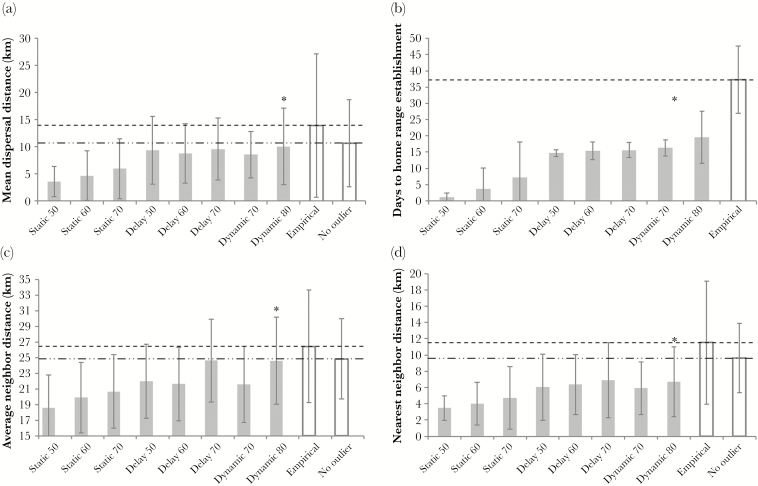
Performance of the best-performing model scenario from each presented configuration based on the ability to match empirical patterns of dispersal by 15 martens translocated to Northern Wisconsin in 2010. Asterisks denote the top performing model overall. Patterns matched that are displayed here include (a) dispersal distance mean and SD, (b) time to home range establishment mean and SD, (c) mean average neighbor distance, and (d) mean nearest neighbor distance. Dashed lines represent empirical means, whereas dashed–dotted lines represent empirical means with one outlier removed (time to home range establishment had no outlier). Means and standard deviations from actual martens with and without a single outlier included are represented by hollow bars. For model simulations, the mean of means and mean standard deviations across 5 replicates are presented. Static and Delay scenarios incorporated a single habitat suitability map with the 50%, 60%, or 70% habitat suitability rule. Delay represents an imposed 2-week exploratory phase prior to settlement. Dynamic scenarios incorporated the 2-week delay as well as a progression of habitat suitability maps over time ([70%, 60%, 50% rules] or [80%, 70%, 60% rules]) to represent a decline in habitat selectivity by the disperser. Bound rates are not displayed here because only the overall best performing bound rate for each scenario is presented.

To test our hypotheses of marten habitat selection, we implemented 3 rounds of the modeling cycle (Grimm and Railsback 2005), building additional complexity into the model after each round (Table 2). First, we tested 3 alternate versions of a static habitat suitability map using a 50%, 60%, and 70% nonavoided habitat type criterion for home range establishment (see Spatial layers: habitat suitability and Figure 2; Table 2). In these scenarios, martens were allowed to establish a home range immediately, with no required exploratory period (Static scenarios). Second, we tested these same scenarios but required individuals to undergo an “exploration phase” of 2 weeks prior to attempting home range establishment (Delay scenarios; Debeffe et al. 2013). We chose 2 weeks because that matched the minimum time to home range establishment from our empirical data set (Figure 3). Third, we implemented a dynamic rule for habitat suitability following Ward’s hypothesis, in which the selectivity criterion for habitat selection was relaxed over time (Dynamic scenarios). Using this dynamic model of habitat suitability, we tested 2 progressions of the selectivity threshold for nonavoided habitat types: (70% to 60% to 50%) and (80% to 70% to 60%; Figure 2; Table 2). In each of these 2 scenarios, the 3 separate home range criteria rules were distributed in equal periods throughout the 60-day dispersal period (i.e., 80% rule for 20 days and then 70% rule for 20 days). For the latter progression, we also tested 2 alternative timing scenarios: one in which the timing for changing habitat suitability maps started immediately, and one in which timing initiation was delayed until after the 2-week exploration phase (Table 2).

**Table 2:** Primary model scenarios tested for their ability to reproduce patterns of dispersal behavior in American martens

Scenario	Habitat selectivity	Percent rule	Exploration phase?	Dynamic habitat timing
Static 50	Static	50	No	NA
Static 60	Static	60	No	NA
Static 70	Static	70	No	NA
Static 50	Static	50	Yes	NA
Static 60	Static	60	Yes	NA
Static 70	Static	70	Yes	NA
Dynamic 70	Dynamic	70, 60, 50	Yes	Immediate
Dynamic 80 (1)	Dynamic	80, 70, 60	Yes	Immediate
Dynamic 80 (2)	Dynamic	80, 70, 60	Yes	Delayed

Percent rules indicate the percentage of habitat in an area required to be a non-avoided cover type in order to be suitable for home range establishment. Higher percentages represent higher selectivity by dispersing martens. Where multiple percentages are listed, this indicates that a dynamic percent rule was used, allowing for habitat selectivity to decline over time. Exploration phase indicates whether martens were required to search for suitable habitat for 2 weeks prior to attempting to establish a home range. Dynamic habitat timing indicates whether the timing of dynamic habitat selectivity was implemented at the start of the simulation (immediate) or following the exploration phase (delayed). Each of these scenarios was replicated 5 times for each of 3 potential movement rates of 10, 20, or 30 bounds per minute.

For each of the 9 scenarios described above (Table 2), we conducted 5 replicates. For each set of replicates, we also tested 3 alternate values for mean rate of movement (MSL) since it was impossible to derive speed of movement from snow-trailing data. This technique, whereby we derived an unknown parameter through model calibration, is known as inverse modeling (Wiegand et al. 2003). We selected 3 distances for MSL that corresponded to an average of 10, 20, or 30 bounds per minute for each sex (McCann et al. 2017). In total, we ran 27 sets of simulations comprising 9 alternative home range establishment criteria crossed with 3 alternative values for MSL (Table 2).

### Data analysis

We used a pattern-oriented modeling approach to evaluate model calibration success (Wiegand 2003; Grimm et al. 2005; Grimm and Railsback 2011). Pattern-oriented modeling requires the identification of a number of observed patterns usually derived from empirical data that are then used collectively as a benchmark to assess model performance. As alternative hypotheses and mechanisms are tested within the IBM, they can each be retained or rejected until a model is identified that successfully matches the selected patterns. This pattern-oriented approach can also be used to calibrate specific uncertain parameters by selecting the parameter values that result in the best model performance (i.e., inverse modeling; Wiegand et al. 2003; Grimm and Railsback 2011). Since complex systems can rarely be described by a single pattern (Grimm et al. 2005), matching multiple patterns increases the likelihood that the model is reproducing the bottom-up (i.e., individual-based) mechanisms driving the system—each pattern acting to filter out poor models and ensure that the best performing model is selected.

We analyzed data collected from translocated individuals that were radio-collared and radio-tracked during dispersal (Woodford et al. 2013) and identified 9 empirical patterns to match as a result of our analysis (Table 1). Seven patterns were matched to an empirical estimate and the other 2 (male to female ratios of dispersal distance and time) were evaluated based on a binary matching criterion (Table 1). Because pattern-matching methods vary widely across studies, we used multiple pattern-matching methods to evaluate which simulation scenarios matched empirical patterns, including pass/fail, weighted pass/fail, rank-sum, weighted rank-sum, Total Indicator (TI) based on root mean square deviation, and a multivariate measure (Mahalanobis Distance, D^2^). Using multiple methods allowed us to assess the level of corroboration for our model selection across a variety of methods used in the pattern-oriented modeling literature, and to assess the effect of the selection of a pattern-matching method on model selection results. To select the best model scenario, we ranked all model scenarios according to each ranking method and summed their totals (Table 3; Supplementary Appendix S2). Not all patterns could be evaluated by each pattern-matching method (Table 1).

**Table 3:** A subset of model ranking results from pattern-oriented modeling

Model rankings									
Scenario	Bounds/ Minute	Exploration Phase	Rank-sum	Weighted Rank-sum	Pass/Fail	Weighted Pass/Fail	Mahalanobis (D^2^)	Total Indicator	Sum
Dynamic 80 (2)	30	Yes	1	1	1	1	4	1	9
Dynamic 80 (1)	30	Yes	2	2	1	1	5	2	13
Dynamic 80 (2)	20	Yes	3	3	3	3	2	3	17
Dynamic 80 (1)	20	Yes	7	9	3	4	6	4	33
Delay 70	30	Yes	5	4	7	4	10	8	38
Delay 70	10	Yes	5	6	7	8	7	7	40
Delay 60	30	Yes	4	5	7	6	12	10	44
Dynamic 80 (2)	10	Yes	12	14	5	13	1	6	51
Static 70	30	No	9	10	5	8	16	11	59
Dynamic 80 (1)	10	Yes	16	16	7	3	3	5	62

Model scenarios shown here were selected as a top 5 model by at least one of the pattern-matching methods. The exploration phase column indicates implementation of a 2-week exploratory threshold preventing individuals from settling. The Percent Rule column indicates the proportion of the area around a cover-type pixel that must be nonavoided to be considered suitable, whether that proportion was static or dynamic (Ward’s prediction) during the simulation, and whether onset of dynamic habitat map swapping was delayed (2) or not (1). Rank-sum ranked each model according to its ability to match each pattern and then summed the resulting ranks. Pass/Fail represents a binary matching criterion and assigned points to a model based on whether a given pattern was successfully matched. TI (Total Indicator) ranked models based on their root mean square deviation from empirical patterns. *D*^2^ (Mahalanobis distance) ranked models based on a multivariate measure that accounts for covariance among patterns. The Sum column indicates the rank-sum for each model across ranking methods. See Supplementary Appendix S1 for full list of model rankings.

We used the pass/fail method to assign points to each simulation scenario based on its ability to match (i.e., pass) each of the 9 patterns. Matching criteria for the numerical patterns were based on 1.96 times the standard error of the mean value from empirical data, as 95% of population means should fall within that range (i.e., 95% confidence interval; Bauduin et al. 2016). We could not calculate empirical standard errors for population-level patterns (i.e., mortality, days to home range establishment SD, and dispersal distance SD); therefore, we bootstrapped empirical data using the “asbio” package in R to estimate 95% confidence intervals and compare with simulation results (Aho 2016; R Core Team 2017). To penalize models that failed to represent differences in dispersal behavior between males and females, we included 2 patterns associated with timing of and distance to home range establishment by sex. To match these patterns of males dispersing farther and taking more time to settle than females, all 5 replicates of a scenario/bound combination had to reproduce the pattern. After all patterns were evaluated, we ranked models based on the number of patterns matched out of 9. We used the same process for the weighted pass/fail method, except that we assigned an importance value of low, medium, or high to each pattern, corresponding with a score of 1, 2, or 3 points (Table 1; Stenglein et al. 2015). After evaluating the models for each pattern, we assigned scores corresponding to the pattern’s importance value which we then summed across all patterns.

Here we describe 4 additional methods used to rank model performance based on pattern-matching. We used the rank-sum method to rank the performance of each of the 27 model scenarios based on their accuracy in reproducing observed patterns (Table 1). We assigned each model scenario a ranking for each of the 7 nonbinary patterns and then summed those rankings across all patterns. The weighted rank-sum method was the same as the nonweighted rank-sum method, except we weighted the model rankings using the same weighting system as described for the weighted pass/fail method (Table 1). The Total Indicator (TI; Piou et al. 2007; Semeniuk et al. 2012) measure was also used to rank model performance. We calculated this metric by calculating the root mean square deviation (RMSD) for each pattern,

$$RMSD=\sqrt{\frac{\sum_{\text{r}=1}^{N_{rep}}{\left(Obs-Sim\right)}^{2}}{N_{rep}}}$$ (1)

where r is the replicate, *N*_*rep*_ is the number of replicates, Obs is the observed mean of a given pattern, and Sim is the simulated mean of the same pattern. We then calculated the TI for each model scenario by summing the ratio of each RMSD to the RMSD of the best performing scenario for a given pattern,

$$TI=\underset{p=1}{\sum^{n}}\frac{RMSD_{p}}{RMSD_{best}}$$ (2)

where *p* represents a given pattern and *n* is the total number of patterns evaluated. Finally, we calculated the mean Mahalanobis distance (*D*^2^; Mahalanobis 1936) across replicates between each model scenario and observed data using the pooled covariance matrix (S; Legendre and Legendre 2012),

$$D^{2}=d_{obs,sim}\cdot S^{-1}\cdot d^{'}{}_{obs,sim}$$

Here *d* represents the vector of differences between the means of the observed and simulated data for each pattern. Unlike the other approaches used, this approach accounts for covariance among patterns. Because *D*^*2*^ uses the covariance matrix, only patterns recorded at the scale of the individual could be evaluated using this metric (Table 1). We ranked models based on *D*^2^ in ascending order.

After pattern-matching and calibration were complete and we had selected our best model, we evaluated that model for its ability to reproduce the general distribution of dispersal distance kernels (k_d_(r); Nathan et al. 2012). This was important because this was a pattern that we selected to be independent of the calibration process. Thus, if our best model produced a classic dispersal distance distribution, this would provide independent evidence that our model was producing realistic dispersal behavior. For martens and across most taxa, dispersal distance kernels are generally leptokurtic (more concentrated around the mean than a normal distribution) and fat-tailed (i.e., positively skewed) due to occasional long-distance dispersal events (Broquet et al. 2006; Nathan et al. 2012). We evaluated our model results for these characteristics by fitting simulated dispersal distances across all replicates from our top model to a Weibull distribution using a maximum likelihood estimator (Paradis et al. 2002). We then conducted the Anderson–Darling Goodness-of-Fit test to measure how well our simulated dispersal distance distribution matched the Weibull distribution. We selected the Anderson–Darling statistic because it gives equal weight to the tails and main body of the distribution (D’Agostino and Stephens 1986).

## RESULTS

We completed 135 simulation runs of 15 dispersing martens each. Of the 9 combinations of home range establishment rules we evaluated, the scenario that included the 2-week delay to home range establishment (i.e., required exploratory phase) and temporally dynamic habitat selectivity (i.e., Ward’s hypothesis) was consistently the top-ranked model across all methods used to evaluate pattern-matching (Table 3). The longer delay in timing of changing the habitat suitability maps (Dynamic 80 (2)) also matched patterns better than the scenario with no delay (Dynamic 80 (1); Table 3). All 6 pattern-matching methods consistently selected the 2-week exploration phase prior to settlement over the same scenarios with no required exploration phase. For bounds per minute of 20 and 30, the dynamic habitat selectivity scenarios performed best (Table 3). However, for 10 bounds per minute, the 70% static habitat scenario performed best (Table 3), although rates of failure to establish a home range were highest under this scenario. Across both dynamic and static habitat selectivity rules, the scenarios with higher habitat selectivity (i.e., more selective martens) matched patterns better than the scenarios with lower habitat selectivity (e.g., 80%, 70%, and 60% rules over the same scenarios with 70%, 60%, and 50% rules; Table 3).

**Figure 4:**
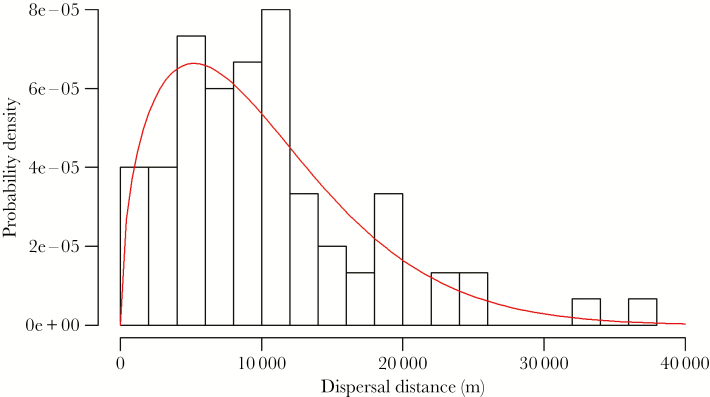
A histogram of dispersal distances across 5 replicates of our top-performing model of American marten dispersal. These data are fitted to a Weibull dispersal distance kernel plotted as the probability density function of the distribution of distances traveled by dispersing martens away from their release location.

Our best-performing model matched 7 of 9 pass/fail patterns, failing to match mean time to home range establishment (Figure 3) and male > female time to settlement (4 of 5 replicates reproduced this pattern). Mean dispersal distance of actual martens (13.9 ± 13.3 km) was 3.8 km greater than simulated martens (10.1 ± 7.1 km), and mean time to establishment of actual martens (37.3 ± 10.3 days) was 17.7 days greater than simulated martens (19.6 ± 8.0 days). One long-distance dispersal outlier (>2 standard deviations from the mean) from the actual martens dispersed 46.7 km, and pattern-matching greatly improved with this outlier removed from the empirical data set (Figure 3). Although our simulations did not produce any dispersal distances greater than 46.7 km, we did record simulated long-distance dispersal events up to 36.6 km (Figure 4). Based on the pass/fail criteria, mean time to establishment was the only pattern not matched by any model scenario. The dispersal distances produced by our model also fit the Weibull distribution (*A*^2^ = 0.40, *P* = 0.84), indicating that the dispersal distance kernel resulting from simulations was both leptokurtic and fat-tailed (Figure 4).

**Figure 5:**
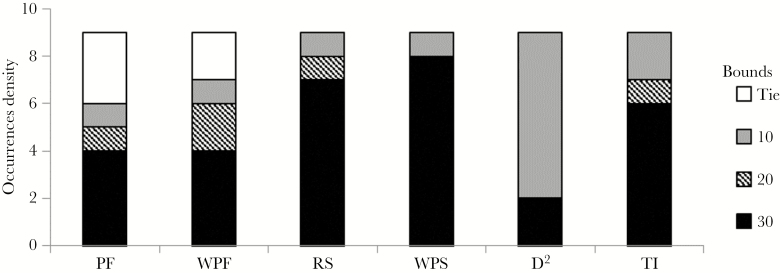
Number of times each of the 3 values tested for number of bounds per minute (i.e., mean step length) was selected as the best performing model by each method used to evaluate pattern-matching. Models simulated American marten dispersal of translocated individuals released in Northern Wisconsin in 2010. PF = Pass/Fail; WPF = Weighted pass/fail; RS = Rank-sum; WRS = Weighted rank-sum; *D*^2^ = Mahalanobis distance; TI = Total Indicator. Several instances occurred in which multiple bound rates received the same ranking from the PF and WPF methods. Two ties occurred between 20 and 30 bounds, 2 ties occurred among all 3 bound rates, and 1 tie occurred between 10 and 30 bounds.

Results from inverse modeling indicated that actual dispersing translocated martens were more likely to move an average of 30 bounds per minute than 10 or 20 bounds per minute in our study area (Figure 5). All pattern-matching methods except *D*^2^ selected 30-bound simulations as the 2 best performing model scenarios (Table 1). *D*^2^ was more likely to select 10-bound simulations as its top performing model scenarios.

## DISCUSSION

We found support for the hypothesis proposed by Ward (1987) that—for a time-limited disperser—the criteria for what constitutes suitable habitat (habitat selectivity) would become less stringent over the course of the dispersal period (Singer et al. 1992; Qian 1999; Withers 1999; Toonen and Pawlik 2001; Elkin and Marshall 2007). To the best of our knowledge, this is the first support for this hypothesis for a behaviorally complex vertebrate. Without this mechanism of dynamic habitat selectivity included, simulations performed poorly at matching empirical patterns of dispersal. For example, when testing static maps with relaxed habitat requirements (i.e., low selectivity), dispersal distances and dispersal times were much shorter than for actual martens. On static maps with high selectivity, dispersers exhibited an unrealistic rate of failure to settle prior to the end of the dispersal period. Thus, allowing individuals to be temporally plastic in their perception of habitat suitability resulted in simulations that best matched empirical data. This mechanism may allow dispersing martens to maximize fitness by undergoing a tradeoff between habitat quality and risk of mortality. Matching empirical patterns also required an imposed 2-week exploratory phase prior to home range establishment. We note here that our support for these hypotheses is based primarily on simulation work, and that additional field work to test our conclusions is an important avenue for further research.

Disperser fitness is directly correlated with home range quality (Powell 2000). Because we concluded that home range selectivity is a function of dispersal time, dispersal time may also be an important indicator of fitness. Others have explored how the timing of home range establishment is affected by factors such as search costs (e.g., mortality risk), body condition, and time available for search (Stamps 2006; Bonte et al. 2012; Travis et al. 2012). Stamps et al. (2005) concluded that longer search times should be accompanied by higher selectivity rather than lower. For example, an individual with more energy reserves should be able to search longer, and thus be more selective. Our simulated martens were not parameterized to respond to increased risk of mortality, began dispersing with equal energy reserves, and were time-limited, so we were unable to test for these effects. Nonetheless, these questions represent additional areas for future empirical and simulation work in the context of Ward’s hypothesis.

Our results suggest that time-limited dispersers experience a tradeoff between habitat quality and risk of mortality. This result may have important implications for the conservation of martens and other solitary dispersers. Martens disperse in the fall and are time-limited because they face starvation and increased exposure to weather and predators if they are unable to locate a home range with suitable resources prior to the onset of winter (Bull and Heater 2001; Johnson et al. 2009). Our results suggest that in poor habitat conditions, martens will disperse farther and for longer periods, ultimately settling for a tradeoff between habitat quality and risk of mortality. Johnson et al. (2009) demonstrated that as natal dispersal distance of martens increased, mortality risk also increased. This effect was increased for martens dispersing through poor habitat and may be further amplified for translocated martens that are unfamiliar with the local environment and the conditions that maximize fitness (Stamps and Swaisgood 2007). This phenomenon may provide some explanation as to why carnivore reintroduction programs have often been unsuccessful (Breitenmoser et al. 2001), since individuals released into inadequate or unfamiliar habitat may have longer search times and are thus subject to greater mortality risk (Stamps and Swaisgood 2007). Such factors should be taken into consideration when planning reintroductions or translocations (Seddon et al. 2007; Pérez et al., 2012).

Overall, our IBM succeeded in reproducing empirical patterns of marten dispersal. In addition to the patterns matched during model calibration, we also tested our output for its ability to reproduce a classic distribution of dispersal distances (Figure 4). It was important to conduct this test after model calibration was complete, because it provided independent verification that our IBM accurately represented the fine-scale mechanisms driving patterns of marten dispersal (Martin et al. 2013). Some deviations from empirical patterns may be attributed to the low frequency of long-distance dispersal events during simulations. Long-distance dispersal can play an important role in a variety of eco-evolutionary processes including population expansion, population connectivity, gene flow, and response to disturbance (Nathan et al. 2012), so it is important to capture long-distance dispersal events. Our simulations did produce long-distance dispersal events (Figure 4) but not at the frequency or distance exhibited by the actual marten population, which may be due to the finite simulation extent. After removing 1 outlier, discrepancies between empirical and simulated patterns were greatly reduced (Figure 3), since it is likely that our small sample of actual marten dispersal distances overrepresented the effect of long-distance dispersal in the empirical data.

The pattern that our models deviated from most was mean time to home range establishment, as simulations resulted in shorter dispersal times than the empirical data. Model output did match the standard deviation of time to home range establishment, however. For management purposes, we were more interested in reproducing spatial patterns of dispersal, since spatial patterns have greater implications for population connectivity, gene flow, and conservation. However, this result also demonstrates that our model may not capture all the fine-scale mechanisms required to accurately reproduce marten dispersal behavior. It is also possible that subjectivity in the analysis of timing of home range establishment affected pattern-matching, as criteria used to define establishment can vary widely in reporting the amount of time individuals explore prior to settlement (McCann 2011; Woodford et al. 2013).

Although pattern-oriented modeling has become standard for the evaluation of ecological IBMs, we demonstrate a need for careful selection of one’s methods for assessing pattern-matching, as methods vary widely across studies [e.g., Stenglein et al. (2015), Bauduin et al. (2016), and Chudzinska et al. (2016)]. Ultimately, individual-based ecology may benefit from an algorithmic framework by which one would select the appropriate method(s) to assess a model’s ability to match empirical patterns (Grimm and Railsback 2011). In our case, we used 6 different techniques (e.g., rank sum and root mean square deviation) to evaluate matching because a consensus across alternative methodologies would provide additional support for the best model. We did identify a consensus in model selection across all 6 methods in terms of habitat selection rules, but the pass/fail methods were less discriminatory than other methods and assigning weights to patterns did not affect the top 3 model rankings (Table 3; Figure 3). In addition, Mahalanobis Distance (*D*^2^) selected a different bound rate from all other methods (though *D*^2^ could only evaluate 4 of 9 patterns). This result likely occurred because *D*^2^ inherently accounts for covariance among patterns (Mahalanobis 1936), and therefore, uncorrelated patterns carry relatively more weight. In our model, the time to home range establishment pattern was the least correlated with all other patterns, and lower bound rates led to longer and more accurate dispersal times. Time to home range establishment was also the one pattern our models failed to match, giving greater weight to better performing scenarios. *D*^2^ and other multivariate statistics may be quite useful in pattern-matching analyses, particularly when researchers want to control for covariance and maintain independence among patterns. In our case, we deliberately chose patterns that were likely to be somewhat correlated to give more weight to the spatial rather than the temporal component of dispersal behavior. Ultimately, the rank-sum and RMSD methods were the most informative for selecting our best model. Thus, careful consideration of factors such as the study’s objectives and the format of the data should be incorporated into the selection of one’s methods for assessing pattern-matching.

In addition to pattern-oriented model selection, we used inverse modeling (i.e., indirect parameterization) to determine that dispersing martens were more likely to move an average of 30 bounds per minute than 10 or 20 bounds per minute (Figure 5). Because our movement data were collected while following marten snow tracks, they lacked a fine-scale temporal component, and therefore, mean step length was an unknown parameter in the model. However, all of our competing model scenarios and pattern-matching analyses except *D*^2^ agreed that the 30-bound version of the model produced the most accurate dispersal patterns. This result aligns well with observed movement rates of a closely related species of marten in North America (Moriarty et al. 2016; Moriarty et al. 2017). The use of pattern-matching to calibrate unknown parameters is often used on a suite of parameters simultaneously [e.g., Kanagaraj et al. (2013)] and is used less often to target a single unknown parameter [e.g., Rossmanith et al. (2007)]. We demonstrate here that when an abundance of empirical data is available for parameterization [e.g., Dumyahn et al. 2007, Gilbert et al. 2009, and McCann et al. (2010)], inverse modeling can be used effectively to derive information about uncertain or unknown parameters related to demographics or behavior (Wiegand et al. 2003).

Our study highlights the power of spatially explicit IBMs to both reproduce empirical patterns and test specific behavioral hypotheses. In our case, we were able to use parameters assigned at a very fine scale (e.g., 15-min time steps) to reproduce patterns at a much larger scale. Such IBMs have been commonly used in both pragmatic (i.e., associated with management goals) and paradigmatic (i.e., associated with underlying theory) contexts (Grimm 1999; DeAngelis and Grimm 2014). Although our model is essentially pragmatic because it addresses a management need for an endangered carnivore, it is paradigmatic in that it explicitly tests a theory of animal behavior. In an early review of ecological IBMs, Grimm (1999) called for an increased focus on theory in individual-based ecology, and we add to that call the need for pragmatic models to test ecological theory produced by both IBMs and traditional ecological models. In this way, pragmatic IBMs can maintain their role as important tools for the management and conservation of wildlife populations (McLane et al. 2011; Wood et al. 2015; Aben et al. 2016), while simultaneously advancing underlying ecological theory (Railsback and Harvey 2002).

Our application of the SEARCH modeling framework can be used in a number of future applications. For example, future work may include pragmatic questions associated with the response of martens to land-use and climate change and to explore the role of long-distance dispersal events in population connectivity. Paradigmatic applications may include the effects of energy reserves and deferred costs on dispersal patterns, which could have important implications for marten populations. Ultimately, researchers do not have to choose whether their IBM application will be entirely pragmatic or paradigmatic, but should take advantage of opportunities to address questions of both management and theory in their use of IBMs in ecology.

## Supplementary Material

Supplementary Appendix S1

Supplementary Appendix S2
