# Supplementary figures and images for: Temporal plasticity in habitat selection criteria explains patterns of animal dispersal
Source: Behav Ecol. 2019 Jan 12;30(2):528–40. doi: 10.1093/beheco/ary193 (PMC6450207; doi:10.1093/beheco/ary193)

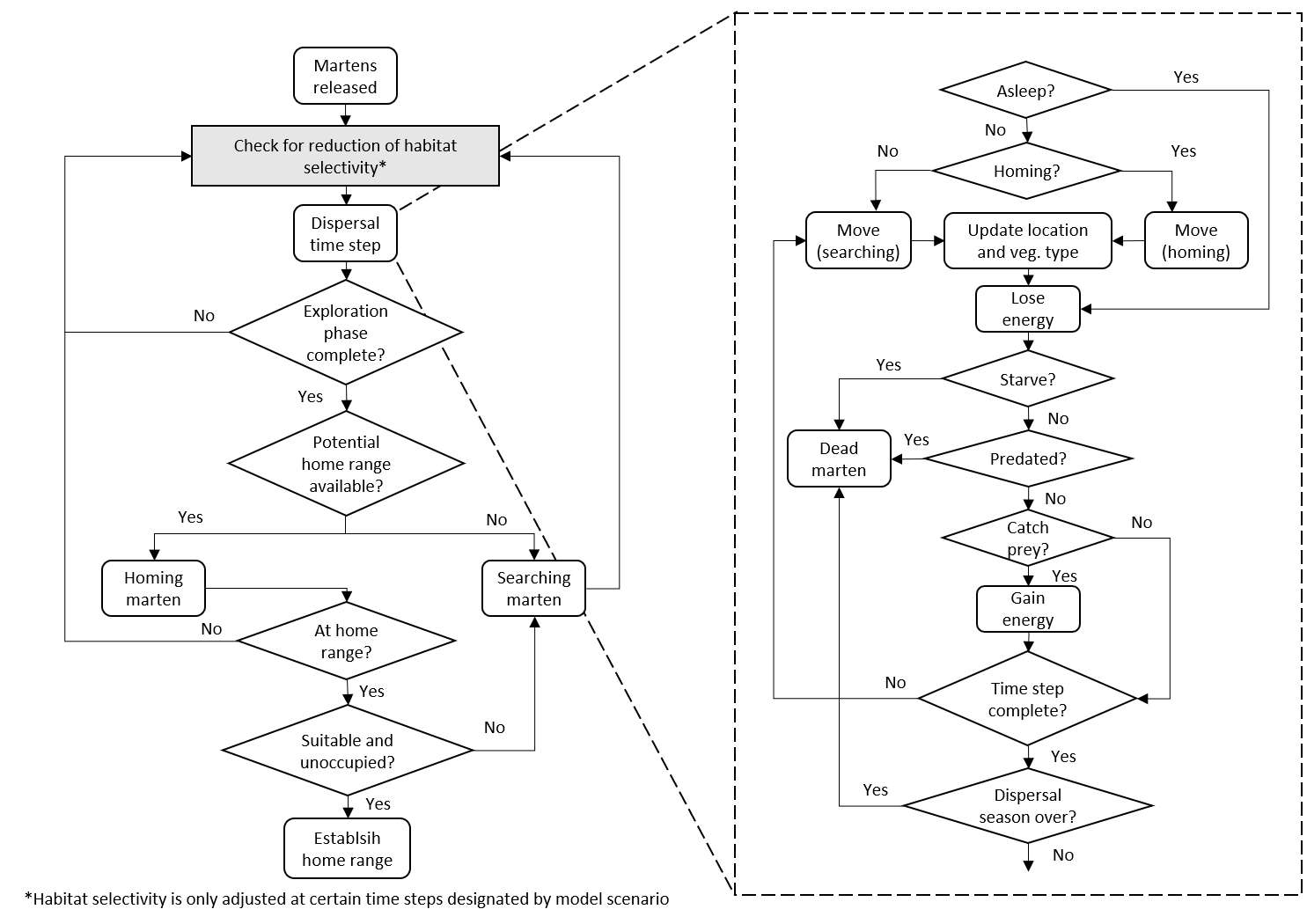

Supplement: Supplementary Appendix S1 [file ary193_suppl_supplementary_appendix_s1.png]
