# Supplementary material for: Temporal plasticity in habitat selection criteria explains patterns of animal dispersal
Source: Behav Ecol. 2019 Jan 12;30(2):528–40. doi: 10.1093/beheco/ary193 (PMC6450207; doi:10.1093/beheco/ary193)
Supplement: Supplementary Appendix S2 [file ary193_suppl_supplementary_appendix_s2.docx]

| Model Rankings | | | | | | | | | |
| --- | --- | --- | --- | --- | --- | --- | --- | --- | --- |
| Delay | Bounds/  Minute | Percent  Rule | **Rank-sum** | **Weighted**  **Rank-sum** | **Pass/Fail** | **Weighted**  **Pass/Fail** | **Mahalanobis Distance** | **Total Indicator** | **Total** |
| Yes | 30 | Dynamic 80 (2) | 1 | 1 | 1 | 1 | 4 | 1 | **9** |
| Yes | 30 | Dynamic 80 (1) | 2 | 2 | 1 | 1 | 5 | 2 | **13** |
| Yes | 20 | Dynamic 80 (2) | 3 | 3 | 3 | 3 | 2 | 3 | **17** |
| Yes | 20 | Dynamic 80 (1) | 7 | 9 | 3 | 4 | 6 | 4 | **33** |
| Yes | 30 | Static 70 | 5 | 4 | 7 | 4 | 10 | 8 | **38** |
| Yes | 10 | Static 70 | 5 | 6 | 7 | 8 | 7 | 7 | **40** |
| Yes | 30 | Static 60 | 4 | 5 | 7 | 6 | 12 | 10 | **44** |
| Yes | 10 | Dynamic 80 (2) | 12 | 14 | 5 | 13 | 1 | 6 | **51** |
| No | 30 | Static 70 | 9 | 10 | 5 | 8 | 16 | 11 | **59** |
| Yes | 20 | Static 70 | 10 | 8 | 17 | 8 | 8 | 9 | **60** |
| Yes | 10 | Dynamic 80 (1) | 16 | 16 | 7 | 15 | 3 | 5 | **62** |
| Yes | 30 | Static 50 | 8 | 7 | 7 | 8 | 18 | 16 | **64** |
| Yes | 20 | Dynamic 70 (1) | 11 | 13 | 7 | 13 | 11 | 12 | **67** |
| Yes | 20 | Static 50 | 15 | 15 | 7 | 6 | 19 | 19 | **81** |
| No | 20 | Static 70 | 18 | 17 | 7 | 8 | 21 | 13 | **84** |
| Yes | 20 | Static 60 | 13 | 11 | 17 | 15 | 17 | 14 | **87** |
| Yes | 30 | Dynamic 70 (1) | 14 | 12 | 17 | 15 | 14 | 15 | **87** |
| No | 10 | Static 70 | 17 | 18 | 7 | 15 | 15 | 17 | **89** |
| Yes | 10 | Dynamic 70 (1) | 19 | 19 | 17 | 21 | 9 | 18 | **103** |
| No | 10 | Static 60 | 23 | 23 | 7 | 15 | 22 | 23 | **113** |
| No | 30 | Static 60 | 20 | 20 | 17 | 20 | 23 | 20 | **120** |
| No | 20 | Static 60 | 22 | 21 | 17 | 22 | 24 | 21 | **127** |
| Yes | 10 | Static 60 | 21 | 22 | 27 | 27 | 13 | 22 | **132** |
| No | 10 | Static 50 | 25 | 25 | 17 | 22 | 25 | 24 | **138** |
| No | 30 | Static 50 | 26 | 26 | 17 | 22 | 26 | 26 | **143** |
| Yes | 10 | Static 50 | 24 | 24 | 26 | 26 | 20 | 25 | **145** |
| No | 20 | Static 50 | 27 | 27 | 17 | 22 | 27 | 27 | 147 |

**Appendix S1.** Complete set of model ranking results from pattern-oriented modeling. Model scenarios shown here were selected as a top 5 model by at least one of the pattern-matching methods. The delay column indicates implementation of a 2-week exploratory threshold preventing individuals from settling. The Percent Rule column indicates the proportion of the area around a cover-type pixel that must be non-avoided to be considered suitable, whether that proportion was static or dynamic (Ward’s prediction) during the simulation, and whether onset of dynamic habitat map swapping was delayed (2) or not (1). Rank-sum ranked each model according to its ability to match each pattern and then summed the resulting ranks. Pass/Fail represents a binary matching criterion and assigned points to a model based on whether a given pattern was successfully matched. TI (Total Indicator) ranked models based on their root mean square deviation from empirical patterns. D^2^ (Mahalanobis distance) ranked models based on a multi-variate measure that accounts for covariance among patterns. The Sum column indicates the rank-sum for each model across ranking methods. See Appendix 1 for full list of model rankings.
